# Spectral-Free Double Light Detection of DNA Based on a Porous Silicon Bragg Mirror

**DOI:** 10.3390/s22187048

**Published:** 2022-09-17

**Authors:** Shuangshuang Zhang, Miao Sun, Xinli Wang, Jiajia Wang, Zhenhong Jia, Xiaoyi Lv, Xiaohui Huang

**Affiliations:** 1 School of Physical Science and Technology, Xinjiang University, Urumqi 830046, China; 2 School of Energy Engineering, Xinjiang Institute of Engineering, Urumqi 830000, China; 3 School of Information Science and Engineering, Xinjiang University, Urumqi 830046, China; 4 The Key Laboratory of Signal Detection and Processing, Xinjiang Uygur Autonomous Region, Xinjiang University, Urumqi 830046, China

**Keywords:** double detection mechanism, porous silicon Bragg mirror, quantum dot, average grey value

## Abstract

To improve the detection sensitivity of a porous silicon optical biosensor in the real-time detection of biomolecules, a non-spectral porous silicon optical biosensor technology, based on dual-signal light detection, is proposed. Double-light detection is a combination of refractive index change detection and fluorescence change detection. It uses quantum dots to label probe molecules to detect target molecules. In the double-signal-light detection method, the first detection-signal light is the detection light that is reflected from the surface of the porous silicon Bragg mirror. The wavelength of the detection light is the same as the wavelength of the photonic band gap edge of the porous silicon Bragg mirror. CdSe/ZnS quantum dots are used to label the probe DNA and hybridize it with the target DNA molecules in the pores of porous silicon to improve its effective refractive index and enhance the detection-reflection light. The second detection-signal light is fluorescence, which is generated by the quantum dots in the reactant that are excited by light of a certain wavelength. The Bragg mirror structure further enhances the fluorescence signal. A digital microscope is used to simultaneously receive the digital image of two kinds of signal light superimposed on the surface of porous silicon, and the corresponding algorithm is used to calculate the change in the average grey value before and after the hybridization reaction to calculate the concentration of the DNA molecules. The detection limit of the DNA molecules was 0.42 pM. This method can not only detect target DNA by hybridization, but also detect antigen by immune reaction or parallel biochip detection for a porous silicon biosensor.

## 1. Introduction

Porous silicon (PS) is widely used in optical biosensor detection because of its large specific surface area, its good biocompatibility, and its easy handling in the preparation of various photonic samples [1,2,3]. In recent years, PS biosensors have been used to detect proteins, cells, enzymes, DNA, and other molecules [4,5,6]. In particular, PS is widely used for DNA detection, because DNA is the most important genetic material constituting cells and it has become a hot topic for biotechnology diagnosis and clinical analysis, due to its inherent molecular recognition properties.

The manufacture and application of photonic crystals is another hot spot in current research. The main characteristic of photonic crystals is that the electromagnetic radiation spectrum changes with periodic changes in the refractive index, so they are widely used in photonics, laser physics, biology, and other fields [7,8,9]. Due to the simple preparation and adjustable optical parameters of PS, a photonic crystal structure can be prepared in PS, which can enhance the transmitted optical signal. A Bragg mirror (BM) can be prepared by adjusting the porosity of PS by changing the preparation parameters [10,11], resonance microcavity [12,13], and rugate filter [14]. These PS optical structures improve the sensitivity of the sensor, due to their band formation and their unique spectral characteristics [15]. When detecting biomolecules using PS photoluminescent biosensors, a BM can amplify the fluorescence intensity changes before and after biological reactions [16].

Previous studies reported that luminophores were embedded into PS photonic crystals, and luminescence enhancement of the luminophores was found. Most of the previously reported luminophores were organic dyes [17,18], but unlike organic dyes, quantum dot dyes (QDs) have the advantages of good light stability, controllable surface properties, a wide excitation spectrum range, and an adjustable emission spectrum [19,20,21]. QDs are water-soluble and biocompatible by surface modification or coupling with functional molecules. After surface modification, QDs can be covalently bonded with biomolecules for biological detection [22]. There are two main ways that QDs are used as markers. The first is to use QDs with unique optical properties to achieve fluorescence enhancement; the second is to use the high refractive index characteristics of QDs to achieve refractive index amplification. Gaur et al. used QD biotin to couple to a streptavidin-fixed PS matrix, and the effective refractive index of PS changed more significantly than that of PS without QDs. QDs amplified the refractive index; the detection limit was reduced by nearly three orders of magnitude; and the detection sensitivity was increased by six times [23].

The detection mechanism of PS optical biosensors is divided into two categories [24,25]. The first category is based on the refractive index change, which is detected by the reflection spectrum or by a Fourier transform spectrum on the surface of the sensor [26,27]. The sensor using this detection mechanism has the advantage of being label-free. The detection limit usually depends on the detection accuracy of the reflection spectrometer, and the equipment cost is high. Zhang et al. used PS as a substrate and egg white lysozyme (HEWL) as an antigen to react specifically with an HCAB heavy chain variable domain (VHH) antibody, resulting in a red shift of the PS reflection spectrum. Different concentrations of the VHH antibody detected had different red shifts of the reflection spectrum. According to the relationship between the two, a detection limit of 0.648 ng/mL was obtained [28]. The second category is based on changes in fluorescence. In terms of biological detection, researchers analyzed the changes in the fluorescence intensity of the reactant’s fluorescent label before and after the biological reaction [29,30]. These sensors are sensitive for detection but require labelling processes, and some of the introduced markers may have an impact on biomolecular interactions [31]. Valerii et al. determined ochratoxin A (OTA) at a low concentration with a photoluminescence immunosensor based on PS. The interaction between PS modified by the anti-OTA antibody and OTA led to PL quenching, and the PL intensity decreased with the increase of Ota concentration, resulting in a detection limit of 4.4 pg/mL [32].

To reduce the detection cost and to improve the detection sensitivity of the biosensor, this paper combines the two detection mechanisms of refractive-index change detection and fluorescence change detection, and proposes a spectral-free double-signal optical detection method based on PS with a Bragg mirror (PSBM). The first signal light in this method is the detection light with a wavelength of 633 nm that is reflected from the PSBM surface. Due to the specific binding of biomolecules in PSBM, the effective refractive index of PSBM increases, while the introduction of QDs amplifies the refractive-index change, resulting in the enhancement of the detected reflected light. The second signal light is the fluorescence emitted by QDs in the bioreactor. QDs in the reactants generate fluorescence with a wavelength of approximately 625 nm under the excitation of short wavelength light. The fluorescence signal was further enhanced by PSBM. The microscope simultaneously receives images of two kinds of light that are superimposed on the PSBM. The difference between the average grey values before and after the reaction was calculated by corresponding algorithms to further detect the target biomolecules. This method not only has the advantages of simple operation and low detection cost, but also high detection sensitivity. This technology can be converted into a small instrument and tested at low cost.

## 2. Theory

A PSBM is a one-dimensional photonic crystal composed of periodically alternating high-refractive index layers and low-refractive index layers, in which the corresponding central wavelength in the photonic band gap can achieve approximately 100% reflectivity, which satisfies the following relationship:

(1) $$\frac{\lambda_{C}}{4}=n_{L}d_{L}=n_{H}d_{H}$$

where *λ_C_* is the center wavelength, *n_L_* and *n_H_* are the refractive index of the low- and high-refractive index layers, and *d_L_* and *d_H_* are the thicknesses of the low- and high-refractive index layers.

The PSBM designed in this paper consists of 24 layers, in which *n_H_* = 1.41, *n_L_* = 1.10, *d_H_* = 98 nm, and *d_L_* = 126 nm. The central wavelength is 553 nm, and the lowest reflectivity is 633 nm at the end edge of the photonic band gap.

The relationship between the effective refractive index and the porosity of PS satisfies the Bruggeman equivalent medium model [33]. Without coupling other molecules, the effective refractive index (neff) can be expressed as:

(2) $$(1-\rho )\frac{n_{Si}^{2}-n_{eff}^{2}}{n_{Si}^{2}+2n_{eff}^{2}}+\rho \frac{n_{air}^{2}-n_{eff}^{2}}{n_{air}^{2}+2n_{eff}^{2}}=0$$

where ρ is the porosity of silicon, *n_Si_* is the refractive index of silicon, *n_eff_* is the effective refractive index, and *n_air_* is the refractive index of air.

According to Equation (2), *n_eff_* decreases with the increase of ρ. We can obtain PS with different porosity by controlling the current density of etching, thereby obtaining a PSBM structure composed of periodic alternation of high and low refractive indices.

**Figure 1:**
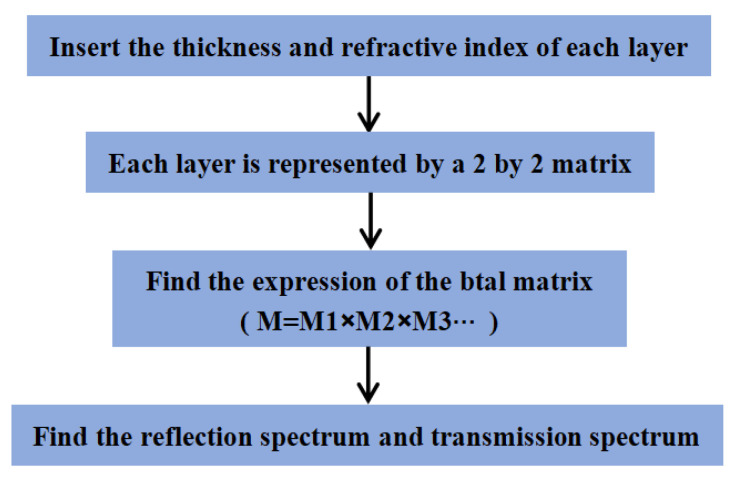
Program structure of the transfer matrix method for a one-dimensional photonic crystal.

Theoretical research on the optical transmission characteristics of photonic crystals is the premise of application research. The theoretical basis of photonic crystals is the classical electromagnetic field theory Maxwell equation. In this experiment, we paid special attention to the transmission coefficient and the reflection coefficient of the incident light field with a certain frequency scattered by the photonic crystal. The transfer matrix (TMM) method is widely used in the design and analysis of optical multilayer thin film structures, as well as photonic crystals [34,35]. The method determines the finite difference solution to Maxwell’s equation in real space and then transforms it into the form of a transfer matrix. Through the transfer matrix, the electric and magnetic fields on one level can be connected with those on another level, so they can be extrapolated to the whole photonic crystal space. The transfer matrix method is used in the design and simulation of PSBM. Figure 1 shows the program structure of the transfer matrix method for a one-dimensional photonic crystal.

**Figure 2:**
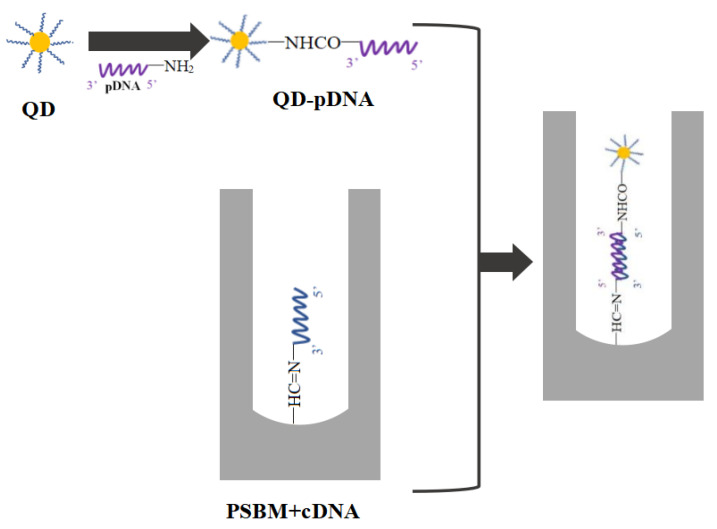
The process diagram of the hybridization reaction between probe DNA successfully coupled to QDs and target DNA in PSBM.

**Figure 3:**
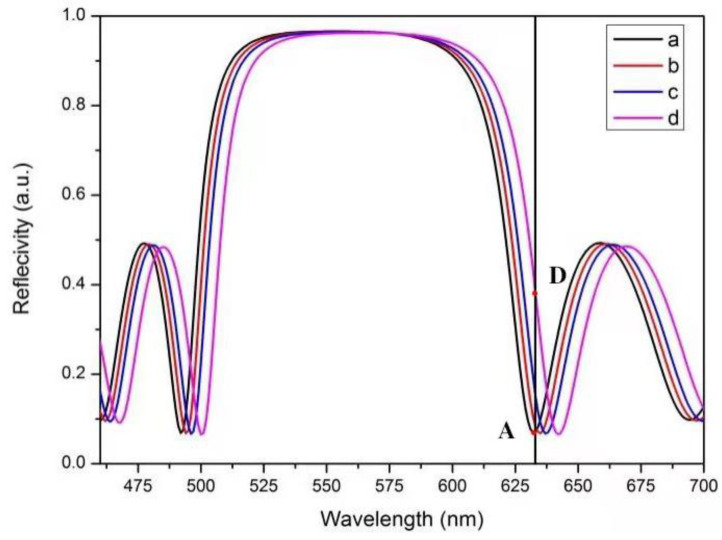
Curve a is the reflection spectrum of the theoretically designed PSBM; curves b, c, and d are the reflection spectra of PSBM after the effective refractive index is increased by 0.005, 0.01, and 0.02, respectively. Point A is the reflectivity at 633 nm of the theoretically designed PSBM, and point D is the reflectivity at 633 nm after the effective refractive index of the PSBM is increased by 0.02. The black line in the figure indicates the reflectivity at the wavelength of 633 nm in the PSBM reflection spectrum.

According to the transfer matrix theory, the reflection spectrum of PSBM is simulated, and the wavelength of the lowest reflectivity at the edge of the photonic band gap is designed to be 633 nm. Assuming that the wavelength of the incident light is 633 nm, the reflectivity of the incident light is the lowest at the edge of the photonic band gap of BM. After the probe DNA molecule is coupled with QDs, it hybridizes with different concentrations of target DNA in the PSBM pores. The hybridization process is shown in Figure 2. As the number of fixed molecules in PS increases, the effective refractive index also increases. The reflectivity at 633 nm at the original band gap edge is the lowest. Due to the red shift of the reflection spectrum, the reflectivity at 633 nm slightly increases, as shown in Figure 3. In Figure 3, b, c, and d are the reflection spectra of PSBM after the effective refractive index is increased by 0.005, 0.01, and 0.02, respectively. It can be seen that the reflectance of point D is significantly higher than that of point A, which shows that the reflected light is enhanced. If QDs with the strongest fluorescence at about 633 nm are used and QDs in the reactant are excited with light of a certain wavelength, it can be concluded that the light intensity on the surface of the PSBM is composed of two parts, as shown in Equation (3):

(3) $$I=I_{R}+I_{Q}$$

where *I* represents the light intensity of the PSBM sample surface, *I_R_* represents the light intensity of the 633 nm probe light on the sample surface, and *I_Q_* represents the light intensity emitted from the QDs from the sample surface.

Therefore, this paper proposes a double-signal light detection method. First, a special PSBM is designed. The edge of the photonic band gap is the same as the wavelength of the detection light, and the wavelength of the fluorescence generated by the label QDs is almost the same. Secondly, the QDs use not only label probe molecules [36], but also generate fluorescence with the same wavelength as the probe light that was excited by light of a certain wavelength. This can achieve refractive index amplification [37]. These two functions doubly enhance the optical signal [38]. Finally, before the biological reaction, the 633 nm detection light is incident on the edge of the PSBM, with the minimum reflected-light intensity and the minimum average grey value of the digital image, denoted as G_1_. After the biological reaction, the effective refractive index of PSBM increases, the photonic band gap shifts to red, and the reflected light is enhanced. QDs in the reactants generate fluorescence under the excitation of the excitation light. The image superimposed by the probe light and the fluorescence light is received by the digital microscope at the same time, and the average grey value, G_2_, is calculated. The variation in the average grey value refers to the difference between the average grey value of the digital image before and after the biological reaction, that is, ΔG = G2 − G1. Thereby, the relationship between the target DNA of different concentrations and the corresponding average grey-value change amount can be obtained, and the concentration detection of DNA molecules can be realized.

**Figure 4:**
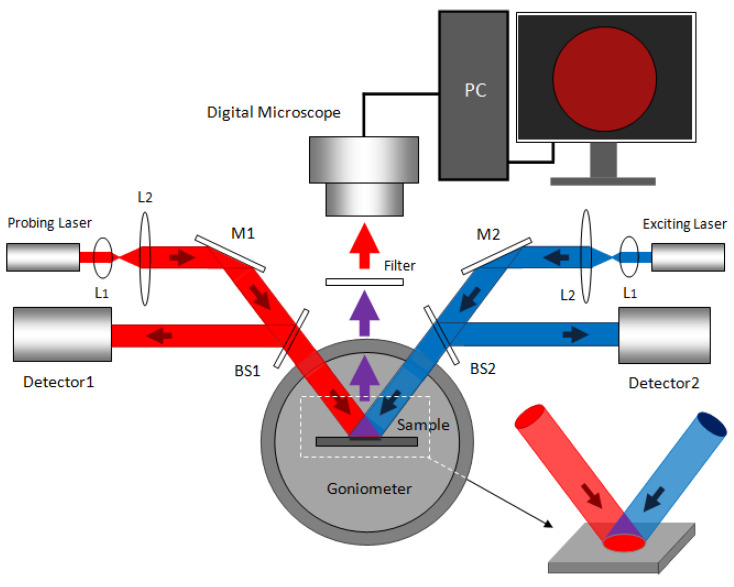
The experimental sample of the PS optical biosensor with different concentrations of DNA by the double-signal optical detection mechanism.

The experimental setup is shown in Figure 4. Light source 1 was the probe light, and an He-Ne laser (λ = 633 nm, 1.8 mW) was used. L_1_ and L_2_ are thin lenses, A stands for the apertures, and M_1_ and M_2_ are the mirrors. They form a collimating and expanding system. A part of the reflected light was detected by detector 1 through the beam splitter, BS. The function of detector 1 was to correct the drift caused by the unstable laser power. Another part of the transmitted light irradiated the PS sample, and the PS was placed in the center of the goniometer. The goniometer was rotated until the brightness was the darkest on the computer; the digital image was received by a digital microscope. The position of the digital microscope was fixed, and the image was displayed on the computer. Light source 2 was an argon ion laser (λ = 488 nm, 60 mW). 

After collimating and expanding through L_1_ and L_2_, the light that was partially reflected after beam splitting was received by detector 2. Firstly, the target DNA was fixed in the hole of the PSBM; then, the probe DNA coupled with QDs was hybridized successfully in the hole of PS, and finally the PSBM was placed in the center of the goniometer. In addition, the surface of the PSBM was irradiated by a 633-nm He Ne laser and a 488-nm argon ion laser, and the goniometer was rotated to the same angle. QDs were excited by 488 nm light to produce approximately 625 nm fluorescence. The PSBM enhanced the luminescence intensity of QDs. The 633-nm filter filtered the excitation light so that the fluorescence generated by QDs and the detection light entered the digital microscope through the filter at the same time, and the image was displayed on the computer. In our detection, the image resolution of the digital microscope was 1600 × 1200; other imaging samples with higher resolution may also be used for image collection. A digital microscope was used to capture and analyze the “grey level” changes. In digital imaging systems, digital microscopy is common, and the received wavelength is usually the visible light wavelength. In this experiment, the received light was red light. If infrared light was detected, an upconversion card was needed [39].

## 3. Experiment

### 3.1. Preparation of the PSBM

The preparation process of PS sample is the same as that in [40].

**Figure 5:**
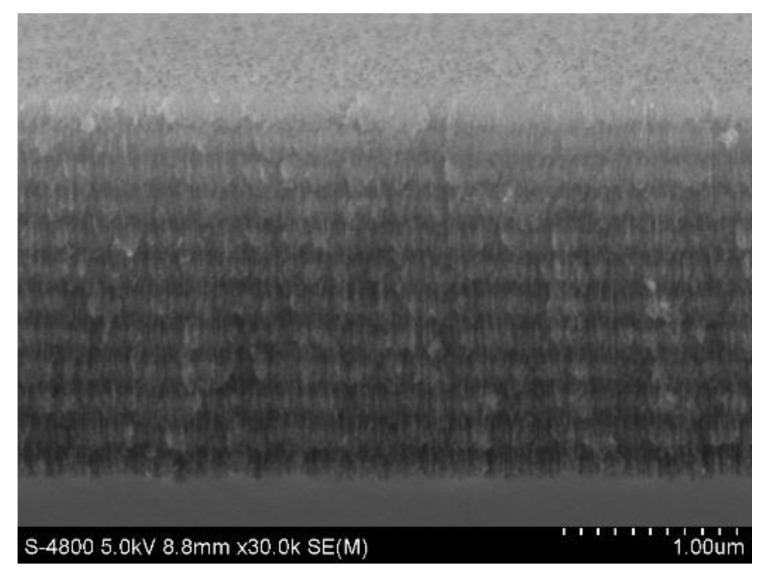
SEM images of cross section of PSBM sample.

**Figure 6:**
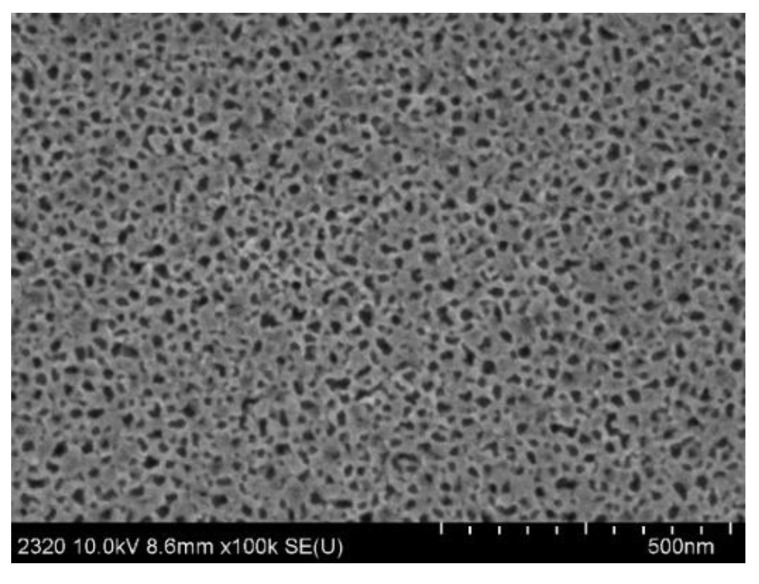
SEM images of surface section of PSBM sample.

From the cross section of Figure 5, it is easy to see that the thickness of the PSBM was 2.6 μm. The surface diagram of Figure 6 shows that the aperture size of the PSBM was approximately 20 nm to 30 nm.

### 3.2. Functionalization of the PSBM

Oxidation treatment, silanization treatment, and glutaraldehyde treatment are the same as the functionalization treatment in [41].

The reflection spectrum was used to test whether the PSBM was functionalized successfully at each step. Figure S1 shows that the reflection spectrum of the oxidation process was blueshifted and that the reflection spectrum of the silanization and glutaraldehyde processes was redshifted. These changes indicated that every step of functionalization in the experiment was successful.

### 3.3. Target DNA Fixed to the Pore Wall of the PSBM

The DNA sequence of 5′-GTTGCAACGTCACATG-3′-NH2 was selected as the target DNA. The target DNA was diluted by PBS to eight concentrations of 1 pM, 5 pM, 10 pM, 20 pM, 40 pM, 50 pM, 70 pM, and 100 pM.

The eight concentrations of the target DNA were fixed on the inner wall of the PSBM, and the experimental steps were the same as those in [40].

### 3.4. Coupling QDs with Probe DNA

**Figure 7:**
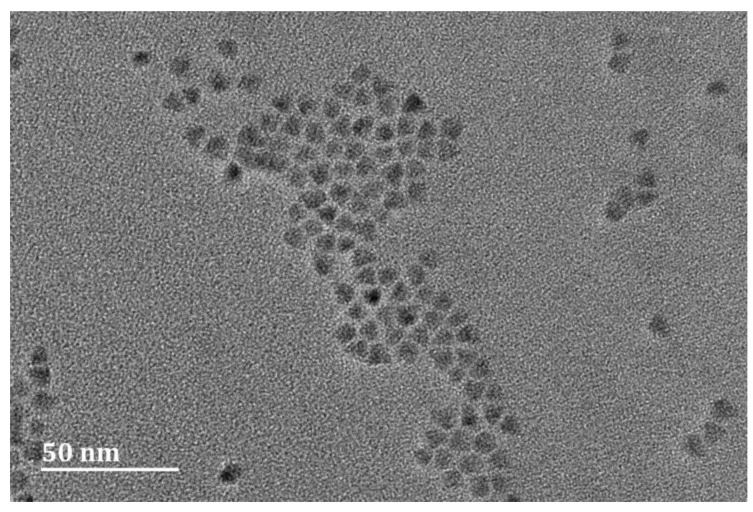
High-power transmission electron microscopy images of water-soluble CdSe/ZnS QDs.

The transmission electron microscopy image of QDs is shown in Figure 7, in which the particle size was approximately 6 nm. According to Figures S2–S7, QDs were successfully coupled to the probe DNA at a concentration of 10 μM. The coupling process of QDs and probe DNA was the same as that in [40].

### 3.5. Hybridization of Target DNA with QDs-pDNA

The experimental process of the hybridization reaction between QDs coupled with probe DNA and target DNA in the PSBM pores was the same as that in [40].

## 4. Results and Discussion

In our experiment, we used a U-4100 ultraviolet visible spectrophotometer to detect the reflection spectra of the target DNA molecules before and after immobilization in PS samples. Figure S8 shows that before the target DNA molecule was fixed, the central wavelength was 555 nm, and the wavelength at the edge of the photonic band gap with the lowest reflectivity was 633 nm. After the 100 pM target DNA was fixed in the pores of the PSBM, the central wavelength of the PSBM was 561 nm and the wavelength at the edge of the photonic band gap with the lowest reflectivity was 636 nm. The results showed that the reflectance spectrum of the PS sample was redshifted after the target DNA molecule was added, which indicated that the target DNA was successfully immobilized in the PSBM.

**Figure 8:**
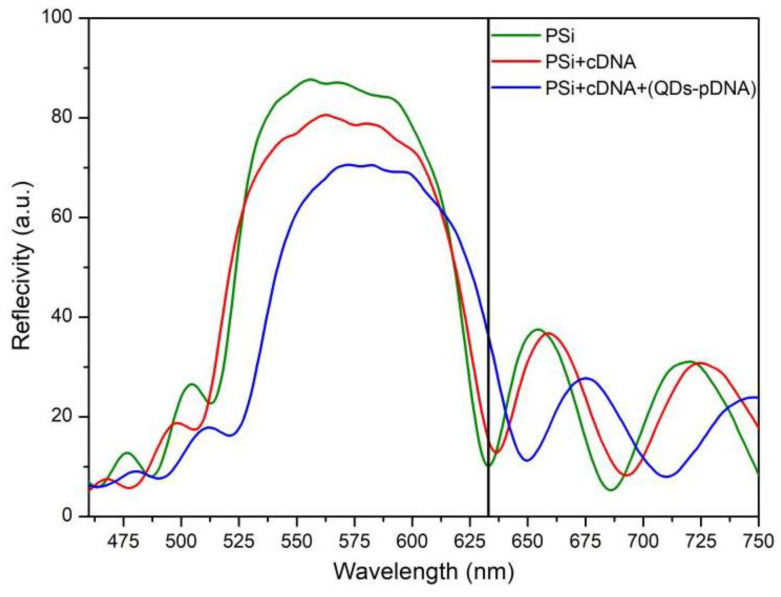
Reflectance spectra of target DNA before and after hybridization with QDs–pDNA. The black line in the figure shows the reflectivity at a wavelength of 633 nm in the reflection spectrum of the BM.

The central wavelength of the high reflection band of the nonhybrid PS samples was approximately 555 nm, and the wavelength range of the high reflection band was from 515 nm to 633 nm. After hybridizing the target DNA with QDs-pDNA, the central wavelength of the high reflection band of the PSBM was 584 nm, and the wavelength range of the high reflection band was from 521 nm to 650 nm. These data show that the DNA molecule was hybridized successfully, as shown in Figure 8.

When the PS sample was prepared, after the functionalization process, the detection light at the edge of PSBM band gap was often inconsistent with 633 nm. During the experiment, when the wavelength corresponding to the lowest edge of the photonic band gap of PSBM was slightly greater than 633 nm, 633 nm was located at the lowest wavelength of the photonic band gap edge of PSBM by adjusting the incident angle of the incident light [42]. When 633 nm detection light was incident on the PSBM surface at a certain angle, the brightness of the digital image was the darkest and was received by the digital microscope. At this time, the average grey value was recorded as G_1_. Eight concentrations of target DNA were hybridized with QDs conjugated probe DNA; a 488-nm argon ion laser was used to irradiate the sample surface of the BM; and the angle of incident light and the irradiation time remained consistent. The samples were imaged by a digital microscope, and the average grey value, G_2_, of the images was calculated. For example, in the PSBM hole, the average grey value of the digital image on the surface of the PSBM before and after the hybridization reaction between 5 pM target DNA and QDs coupled with probe DNA was 17.95 and 20.57, respectively, and the average grey value of the digital image on the surface of PSBM before and after the hybridization reaction between 20 pM target DNA and QDs coupled with probe DNA was 13.78 and 20.74, respectively.

In the grey analysis of the digital image, the corresponding algorithm was used to calculate the average grey value of the grey area in the digital image. The region was a circular region; its center coincided with the center of PS, and its diameter was approximately two-thirds of the diameter of the circular digital image. To reduce the calculation error, the same circular region was selected in the digital images of different concentrations of target DNA. The average grey-value changes. ΔG, before and after hybridization of eight concentrations of target DNA and probe DNA coupled to QDs, were 1.10, 2.16, 3.69, 6.96, 10.98, 13.52, 14.19, and 15.25, respectively.

**Figure 9:**
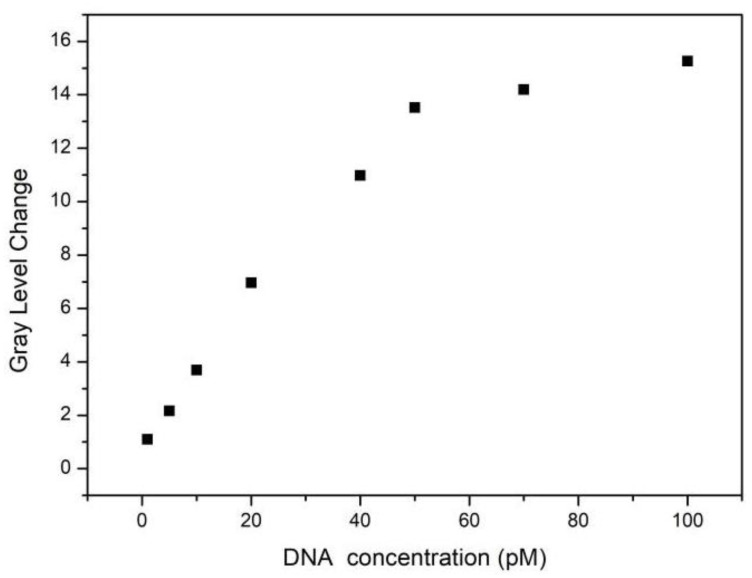
Eight concentrations of target DNA with the corresponding average grey-value change trend.

**Figure 10:**
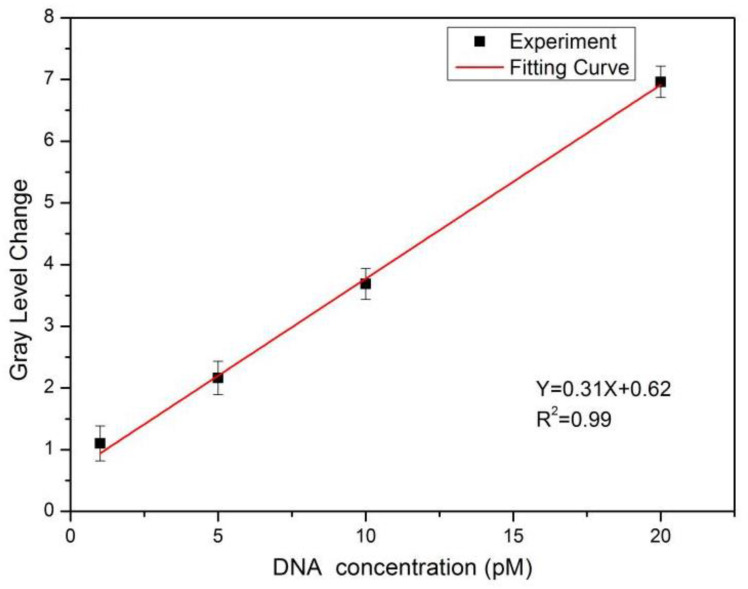
Linear relationship between the target DNA at 1 pM, 5 pM, 10 pM, and 20 pM and the change in the average grey value.

The trend diagram of the target DNA of eight concentrations and its corresponding average grey-value change is shown in Figure 9. With the increase in the DNA concentration in the PSBM, the change in average grey value also increased. The DNA molecules were between 1 pM and 100 pM, and the change in average grey value increased with the increase in the concentration of the added biomolecules, which had a nonlinear relationship as a whole. The DNA molecules were between 1 pM and 50 pM, and the change of average grey value was almost linear with the concentration of the added DNA molecules. Among the eight concentrations of target DNA, four low concentrations of the target DNA were selected to fit the detection limit of the PSBM biosensor. As shown in Figure 10, the fitting coefficient was 0.99, and the linear equation was Y = 0.31X + 0.62.

The surface undulation of PS caused accidental errors in the process of the experiment, which affected the detection accuracy. The 3σ rule was used to calculate the experimental results, where 3σ is the minimum resolvable grey value and σ is the standard deviation of the average grey value of the same PSBM (without biomolecules) measured 10 consecutive times; that is, after measuring the digital image of the PS sample before 10 biological reactions and calculating the corresponding average grey-level value, the value of the experimental detection, σ, was 0.25. The minimum value change of the average grey value of the digital image was 0.75. As shown in Figure 10, the detection limit was 0.42 pM.

The stability and reproducibility of the sensing performance can detect the target molecules more accurately [43,44]. To test the reproducibility and stability of the PS optical biosensor, five PSBMs with the same optical properties were prepared under the same preparation conditions (doping type, resistivity, orientation, and etching conditions). In this paper, the reproducibility of the biosensor detected 100 pM DNA molecules by using this method. The relative standard deviation of the detection results was 4.85%. To determine the stability of the PS sensors, five PSBM sensors were used to detect the corresponding digital images every five days, and a relative standard deviation of 6.1% was obtained. The results showed that the PS biosensor had good reproducibility and stability.

For the labelled PS biosensor, QDs were used to label the biomolecules to achieve refractive-index amplification. The angle spectrum method, combined with QDs, was used to measure the incident angle of the minimum reflected-light intensity before and after the DNA hybridization reaction in the PS microcavity biosensor. The detection limit of the DNA was 36 pM [41]. To detect the fluorescence changes of probe molecules labelled with QDs, the fluorescence images of 20 base pairs of the target DNA and the QD-modified probe DNA in the PSBM before and after hybridization were obtained by the imaging method and with digital- imaging equipment, and the average grey-value change before and after the hybridization reaction was obtained. The detection limit of target DNA was 88 pM [40]. Compared with [40], the difference was that the optical properties of the PSBM photonic band gap edge were used to detect 16 base pairs of DNA molecules. Compared with [41], the difference was that the PSBM was used in this experiment, and the digital image method was used for detection. In the case of different PS samples, detection methods, and biomolecules, the double-optical signal detection method proposed in this paper has higher detection sensitivity than the above two detection methods.

In the experiment, we tested whether the combination of the two detection methods improved the sensitivity and reduced the detection limit, compared with the single-detection method. Using the detection sample shown in Figure 4, the change in the average grey value before and after the hybridization reaction was greater than the change when only the refractive-index change detection method or the fluorescence-change detection method was used. Single-method detection is realized by blocking the probe light or the excitation light, and the average grey-value change in the refractive-index change measurement method or the fluorescence-change measurement method can be obtained. In addition, because this method does not require spectrometer detection and the detection cost is very low, it can not only detect target DNA by DNA hybridization, but also detect antigen by means of immune reaction.

## 5. Conclusions

In this paper, a new mechanism of double-light detection of DNA based on PS biosensor was proposed. The first signal was the 633 nm emitted light reflected from the surface of the PSBM. After labelling with semiconductor QDs, the bioprobe reacted with the target molecule in the PSBM, and the semiconductor QDs enabled refractive-index amplification. Due to the specific binding of biomolecules in the PSBM, the refractive index of the sample increased, resulting in enhanced detection of reflected light. The second signal was the fluorescence of the QDs in the bioreactor. The QDs in the reactant were excited by a 488 nm laser, and the fluorescence wavelength was approximately 625 nm. The fluorescent signal was enhanced by the PSBM. The corresponding algorithm was used to calculate the average grey value of digital image before and after adding DNA biomolecules. The DNA concentration of the target was detected by the change in the average grey value, and the detection limit was 0.42 pM. This mechanism can greatly improve the sensitivity of the PSBM biosensor, which can be used for parallel biochip detection of the PS biosensor, and for antigen detection by immune reaction.

## Supplementary Materials

The supporting information can be downloaded at: https://www.mdpi.com/article/10.3390/s22187048/s1. Figure S1: Reflection spectrum comparison of the PSBM after each step of functionalization. Figure S2: EDS-TEM spectra of CdSe/ZnS QD-COOH. Figure S3: Fluorescence spectra of QDs excited by four wavelengths of excitation light. Figure S4: XPS spectrum of QDs. Figure S5: XPS spectrum of the probe DNA molecules coupled with QDs. Figure S6: Fourier transform infrared spectroscopy of DNA molecules coupled with QDs. Figure S7: Fluorescence spectra of QDs and 10 μM probe DNA before and after coupling. Figure S8: Comparison of reflectance spectra of target DNA before and after immobilization in PSBM hole.
