# Supplementary material for: Spectral-Free Double Light Detection of DNA Based on a Porous Silicon Bragg Mirror
Source: Sensors (Basel). 2022 Sep 17;22(18):7048. doi: 10.3390/s22187048 (PMC9503906; doi:10.3390/s22187048)
Supplement: Supplementary file 1 [file sensors-22-07048-s001.zip › sensors-1866040-supplementary.pdf]

Supplementary material

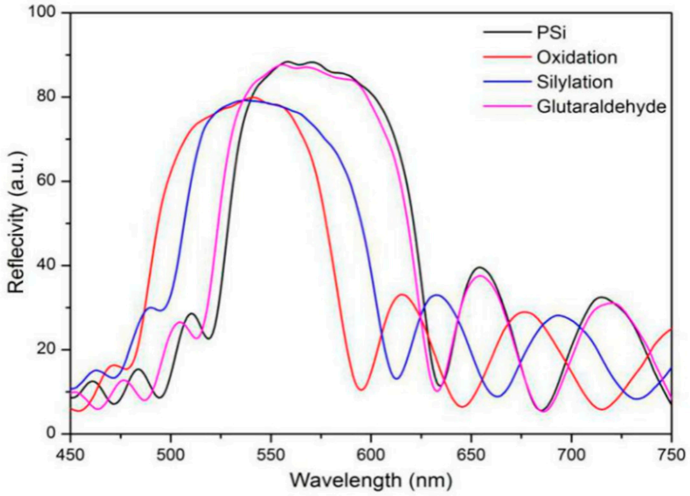

Figure S1. Reflection spectrum comparison of the PSBM after each step of functionalization.

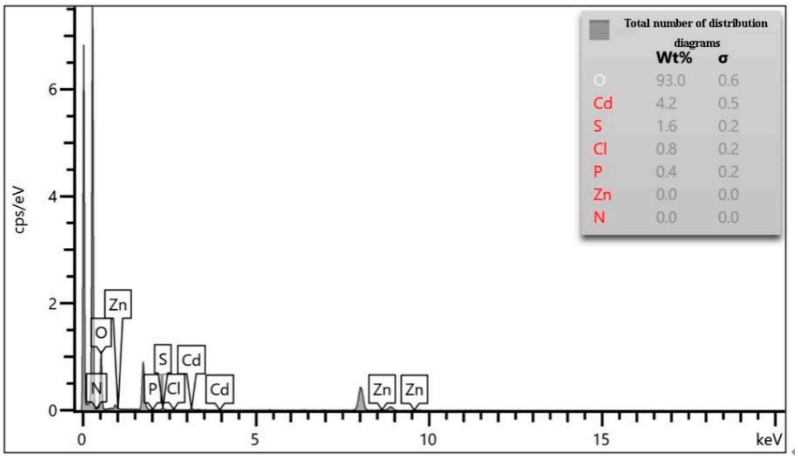

Figure S2. EDS-TEM spectra of CdSe/ZnS QD-COOH.

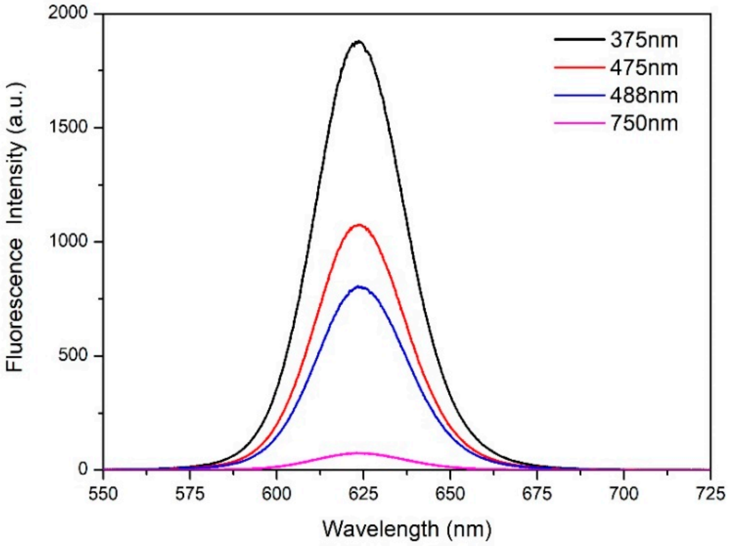

Figure S3. Fluorescence spectra of QDs excited by four wavelengths of excitation light.

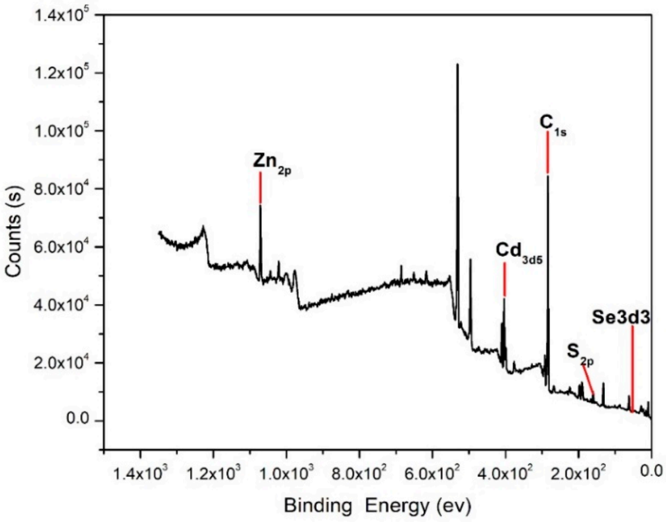

Figure S4. XPS spectrum of QDs.

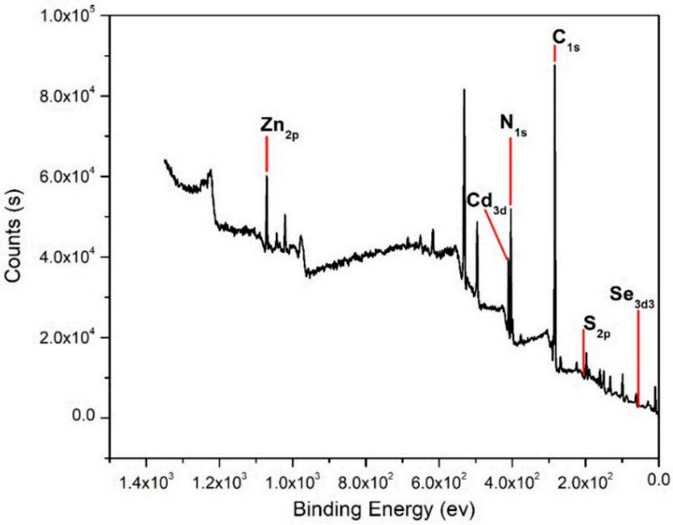

Figure S5. XPS spectrum of the probe DNA molecules coupled with QDs.

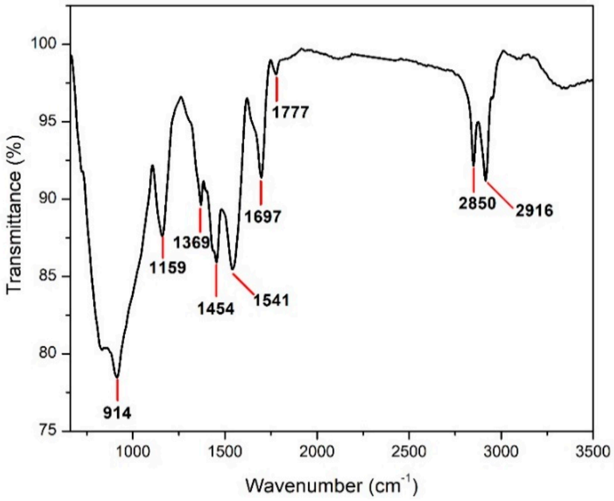

Figure S6. Fourier transform infrared spectroscopy of DNA molecules coupled with QDs.

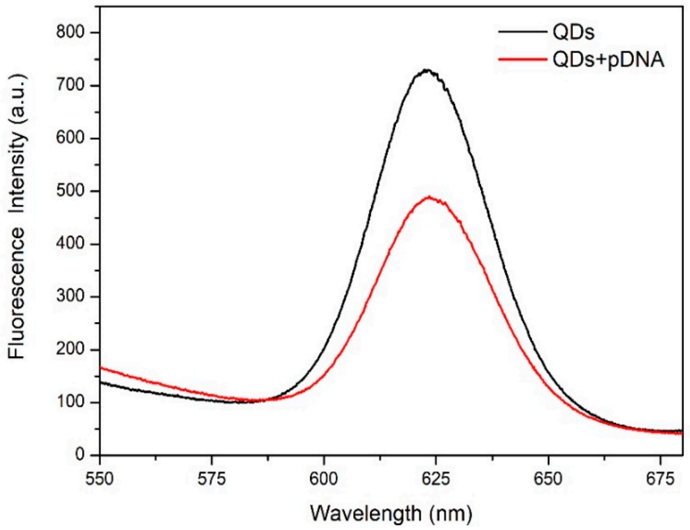

Figure S7. Fluorescence spectra of QDs and 10μM probe DNA before and after coupling.

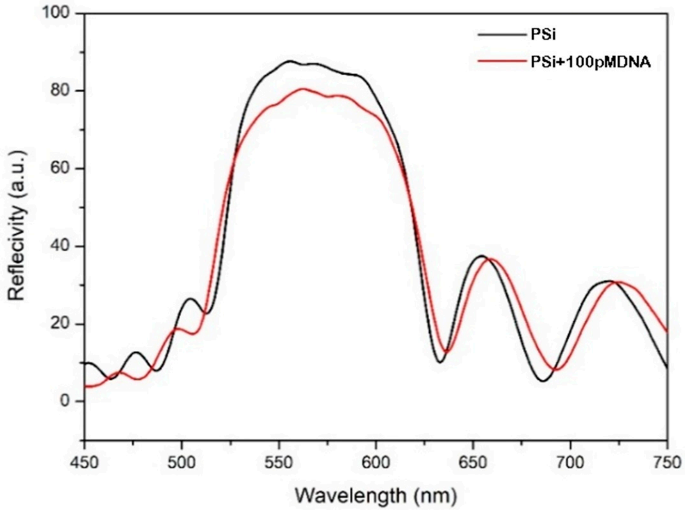

Figure S8. Comparison of reflectance spectra of target DNA before and after immobilization in PSBM hole.
